# Inflammation and Cardiovascular Diseases in the Elderly: The Role of Epicardial Adipose Tissue

**DOI:** 10.3389/fmed.2022.844266

**Published:** 2022-02-15

**Authors:** Maddalena Conte, Laura Petraglia, Paolo Poggio, Vincenza Valerio, Serena Cabaro, Pasquale Campana, Giuseppe Comentale, Emilio Attena, Vincenzo Russo, Emanuele Pilato, Pietro Formisano, Dario Leosco, Valentina Parisi

**Affiliations:** ^1^Department of Translational Medical Sciences, University of Naples Federico II, Naples, Italy; ^2^Casa di Cura San Michele, Maddaloni, Italy; ^3^Centro Cardiologico Monzino IRCCS, Milan, Italy; ^4^Department of Advanced Biomedical Science, University of Naples Federico II, Naples, Italy; ^5^Department of Cardiology, Monaldi Hospital, Naples, Italy; ^6^Department of Medical Translational Sciences, Monaldi Hospital, University of Campania Luigi Vanvitelli, Campania, Italy

**Keywords:** elderly, inflammation, epicardial adipose tissue, cardiovascular diseases, coronary artery disease, aortic stenosis, atrial fibrillation, heart failure

## Abstract

Human aging is a complex phenomenon characterized by a wide spectrum of biological changes which impact on behavioral and social aspects. Age-related changes are accompanied by a decline in biological function and increased vulnerability leading to frailty, thereby advanced age is identified among the major risk factors of the main chronic human diseases. Aging is characterized by a state of chronic low-grade inflammation, also referred as inflammaging. It recognizes a multifactorial pathogenesis with a prominent role of the innate immune system activation, resulting in tissue degeneration and contributing to adverse outcomes. It is widely recognized that inflammation plays a central role in the development and progression of numerous chronic and cardiovascular diseases. In particular, low-grade inflammation, through an increased risk of atherosclerosis and insulin resistance, promote cardiovascular diseases in the elderly. Low-grade inflammation is also promoted by visceral adiposity, whose accumulation is paralleled by an increased inflammatory status. Aging is associated to increase in epicardial adipose tissue (EAT), the visceral fat depot of the heart. Structural and functional changes in EAT have been shown to be associated with several heart diseases, including coronary artery disease, aortic stenosis, atrial fibrillation, and heart failure. EAT increase is associated with a greater production and secretion of pro-inflammatory mediators and neuro-hormones, so that thickened EAT can pathologically influence, in a paracrine and vasocrine manner, the structure and function of the heart and is associated to a worse cardiovascular outcome. In this review, we will discuss the evidence underlying the interplay between inflammaging, EAT accumulation and cardiovascular diseases. We will examine and discuss the importance of EAT quantification, its characteristics and changes with age and its clinical implication.

## Introduction

Life expectancy has improved and, therefore, the proportion of older individuals in the general population is increased. These demographic changes led to a considerable increase in the prevalence of chronic diseases, such as cardiovascular diseases, a major public health problem (1, 2).

Aging is characterized by a chronic low-grade proinflammatory state that results in a progressively greater susceptibility to multimorbidity, disability, and death. This pro-inflammatory status, a condition often named inflammaging, is promoted by sustained high levels of pro-inflammatory markers (3–5).

Inflammaging represents a risk factor for cardiovascular diseases and is associated to adverse outcomes in the elderly (6, 7). It has been recognized a potential role of visceral adipose tissue (VAT) in inflammaging. The accumulation of visceral fat depots is accompanied by an increased production and secretion of inflammatory mediators. Accumulation of abdominal VAT is closely associated with increased prevalence of insulin resistance and metabolic syndrome, and it is related to an increased risk of worse cardiovascular outcomes (8).

Accumulating evidence strongly support the role of structural and functional changes of epicardial adipose tissue (EAT), the visceral fat depot of the heart, in the pathogenesis of various cardiovascular diseases. Interestingly, a greater EAT amount is observed in the elderly. EAT is a metabolically active tissue that in pathological conditions assumes a pro-inflammatory phenotype. EAT-derived pro-inflammatory mediators and neuro-hormones act on the myocardium and coronary vessels in a paracrine and vasocrine manner, thus contributing to development and progression of cardiovascular diseases (9).

Different imaging methods, such as echocardiography, computed tomography (CT) and cardiac magnetic resonance (CMR), have been used for the quantification of EAT in clinical research studies. Given the association between EAT with the onset and progression of cardiovascular disease and its impact on cardiovascular outcome, EAT measurement has been proposed as useful marker for assessment of cardiovascular and metabolic risk (10). However, there is still a lack of consensus on how to measure EAT in clinical practice.

**Figure 1:**
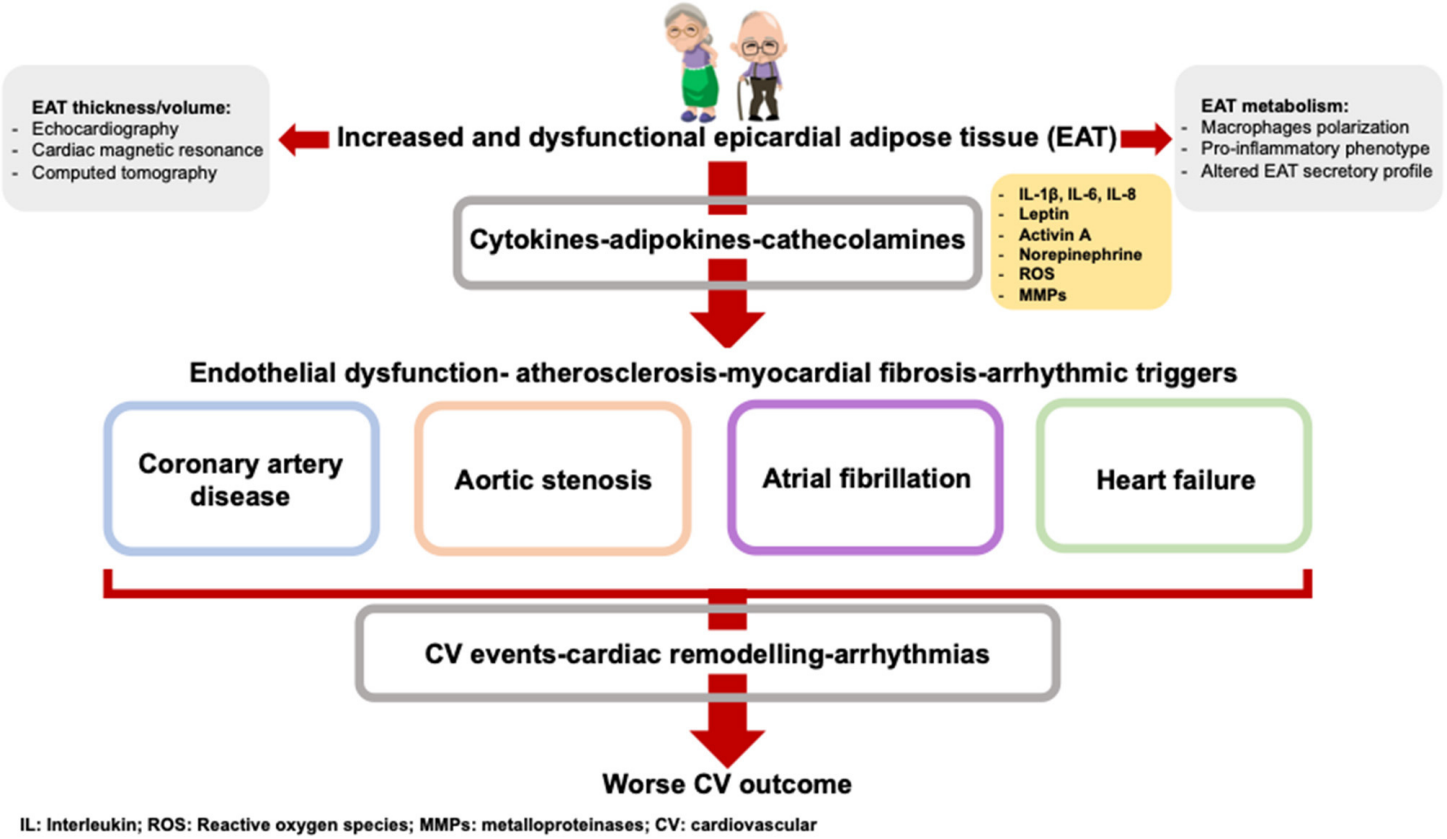
The age-related EAT accumulation is paralleled by an EAT altered metabolism. The adipokines produced by EAT contribute to the onset and the worse prognosis of cardiovascular diseases.

In this review, we will discuss the evidence underlying the interplay between inflammaging, EAT accumulation and cardiovascular diseases. We will examine and discuss the characteristics and changes of EAT occurring with age and its clinical implication (Figure 1). Finally, we will describe the different available image methods to measure EAT amount and we will emphasize the importance of EAT quantification in assessing cardiovascular and metabolic risk.

## Aging and Inflammation

Chronic inflammation is a typical feature of aging. Therefore, the term inflammaging has been introduced to describe the low-grade, chronic, systemic, and sterile inflammation that occurs without overt infection, and results in progressive tissue damage and degeneration. This proinflammatory state is characterized by high circulating levels of proinflammatory mediators, including interleukin (IL)-1, IL-6, IL-8, IL-18, C-reactive protein (CRP), interferon (IFN)-α and IFN-β, growth factor transformant-β (TGF-β), tumor necrosis factor (TNF) and serum amyloid A, even in the absence of risk factors and clinically active diseases (11).

Inflammation, which represents an important defense mechanism against infections or foreign molecules, becomes particularly harmful when it is sustained and prolonged. Thus, inflammaging is recognized as a determinant of adverse health outcomes, leading to a higher risk of morbidity and mortality in older people.

The etiology of inflammaging is very complex and heterogeneous, including genetic factors, cellular senescence, immunosenescence, age-related changes in coagulation and gut microbiota (12).

Genetic polymorphisms can promote a pro-inflammatory state with aging, leading to the upregulation of immune and inflammatory pathways and influencing the circulating levels of various inflammatory markers, such as IL-6 (13).

Several evidence suggest that age-related changes in microRNAs (miRNAs) could contribute to inflammaging. miRNAs are non-coding, single-stranded RNAs, that play important roles in regulating gene expression. Significant differences have been described in the specific miRNAs levels in elderly circulating cells, plasma and whole blood compared to younger subjects, with over-representation of miR-21–5p and miR-126–3p and under-representation of miR-25–3p, miR-92a-3p, miR-93–5p, miR-101–3p, miR-106b-5p, miR-142–5p, miR-151a-3p, and miR-181a-5p. Age-related changes in miRNAs can affect cellular senescence and modulate immune responses, thus promoting the low-grade inflammation state (12, 14, 15).

Among the identified major sources of inflammation in the elderly, there is the imbalance between production and elimination of host-derived endogenous cell debris. The accumulation of biological debris with age support chronic inflammation, by miming bacterial products and activating innate immunity. In these conditions, cellular and organelle components, free radicals and metabolites are recognized as harmful by a network of sensors, including the NOD-, LRR- and pyrin domain-containing protein 3 (Nlrp3) inflammasome, and activate the immune reactions for physiological repair. However, over time, such responses can become chronic and therefore maladaptive (16).

The multiprotein complex of Nlrp3 inflammasome acts in response to cellular danger through the activation of procaspase-1, with consequent production and secretion of proinflammatory cytokines IL-1β and IL-18. Among the activators of the Nlrp3 inflammasome, there are both pathogen-associated molecular patterns and endogenous host-derived molecules indicative of cellular damage, that act mainly through the production of mitochondrial reactive oxygen species (ROS). Furthermore, the mitochondrial cardiolipin, in the case of mitochondrial dysfunction, can act as endogenous pathogen-associated molecular pattern and as binding site for Nlrp3, capable of activating the Nlrp3 inflammasome proinflammatory pathway (17).

Cellular senescence, the normal cellular response to damage and stress, is another important determinant of inflammaging. On the one hand, it suppresses the proliferation of genotypically damaged cells, preventing cancer, and contributes to the wound healing process. On the other hand, senescent cells accumulate with age in many tissues and through their secretory profile (secretory phenotype associated with senescence or SASP), they promote age-related disease by altering the structure and function of different organs. SASP consists in a wide range of soluble molecules, including IL-1α, IL-1β, and IL-6, chemokines (IL-8 and growth-regulated-α protein), growth factors (fibroblast growth factor 2 and hepatocyte growth factor), metalloproteinases (MMP1, MMP3, and MMP13), and other insoluble proteins and extracellular matrix components. These secretory mediators act in a paracrine manner, influencing neighboring cells, but can be also released in systemic circulation, thereby strongly contributing to inflammaging. Interestingly, a marked accumulation of senescent cells has been described in the visceral fat of obese individuals, that is an important source of inflammatory cytokines (18, 19).

The VAT, usually defined as abdominal accumulation of adiposity, mainly localized at the omental and mesenteric level, is composed not only of adipocytes, but also of other cells, such as the stromal vascular fraction of fibroblasts, endothelial cells, macrophages and preadipocytes (20).

Accumulation of abdominal VAT is closely associated with increased prevalence of insulin resistance and metabolic syndrome, and it is related to an increased risk of cardiovascular outcomes (8, 21).

The persistent positive caloric balance, as it occurs in obesity, induces an enlargement and consequent metabolic and immune dysfunction of the adipocytes. The increase in visceral fat correlates to a proatherogenic alteration of the lipid profile, with a reduction of high-density lipoproteins and an increase in small low-density lipoprotein particles (LDLs) (22). Pathogenetic mechanisms underlying the complications related to visceral obesity include the activation of lipolysis and production of free fatty acids (FFAs), increased oxidative stress, adipocyte hypoxia and apoptosis (23). Furthermore, the monocyte infiltrate turns toward an inflammatory sense with generation of M1 macrophages, that lead to increased secretion of proinflammatory cytokines, which contribute to a chronic inflammatory state (24).

The age-related changes of the immune system, also named immunosenescence, also contributes to inflammaging. With aging, in fact, an immune dysregulation with a reduction in adaptive immunity and a hyperactivity of innate immunity has been described. In the elderly, CD4 + lymphocytes show greater intrinsic activation of the nuclear factor-κB (NF-κB) pathways than in younger individuals. These age-related changes are promoted by genetic factors, by intrinsic cellular changes of the immune system and by permanent exposure to antigens and pathogens, and can be accelerated and aggravated by persistent infections, sustained by some viruses, such as Epstein–Barr virus and Cytomegalovirus (25).

Another source of inflammation seems to be linked to age-related changes in the gut microbiota, capable of inducing an inflammatory response through the production of harmful products and metabolites. This process seems to be favored by the age-related reduced intestinal ability to sequester pathogenic microbes and their products. Age-related gut microbiota changes consist, on the one hand, in a decrease in beneficial commensal microorganisms, such as Coprococcus, Faecalibacterium, and Lactobacillus, which counteract the development of microbial pathogens and maintain the integrity of the intestinal barrier; on the other hand an increase in anaerobic pathogenic bacteria—such as Fusobacterium and Staphylococcus—occur, associated with an increase in the mucosal barrier permeability, thereby allowing bacteria and their products into the circulatory system. These factors contribute to increased plasma levels of inflammatory cytokines, thus sustaining the chronic pro-inflammatory state (26).

Overall, the possible sources of inflammation are various and very heterogeneous. Probably, these different mechanisms are interconnected and act synergistically with different relevance and combinations in selected individuals.

## Inflammation and Cardiovascular Diseases

The role of inflammation in the pathophysiology of several cardiovascular diseases is widely recognized and its involvement in the pathogenesis and progression of atherosclerotic processes has already been hypothesized many years ago, based on autopsy studies which showed the presence of abundant inflammatory infiltrates in the adventitia of the coronary arteries of patients who died for acute coronary syndrome (27).

The close association between inflammation, immune response and cardiovascular diseases has been then supported by several experimental data, that identified immune cells and inflammatory mediators involved in atherogenesis. The binding and penetration of lymphocytes into the vascular endothelium, the proliferation and migration of smooth muscle cells toward the intima of the vessel wall, the transendothelial recruitment of macrophages have been described as key events in the formation of atherosclerotic plaque (28).

Subendothelial accumulation of LDLs, the increased generation of ROS, and the consequent LDLs oxidation by ROS are associated with endothelial injury and dysfunction, thereby promoting the onset and progression of atherosclerotic process. Oxidized LDL induce endothelial cell expression of adhesion molecules, such as vascular cell adhesion molecule-1 (VCAM-1), intercellular adhesion molecule 1 (ICAM-1), that bind monocytes and T lymphocytes, allowing them to penetrate the intima. In addition, chemoattractant mediators such as complement factors and monocyte chemoattractant protein-1 (MCP-1), are responsible for recruitment into the vascular wall of mononuclear cells, that differentiate into macrophages, ingest oxidized LDLs, and become foam cells. Interestingly, activated T cells and macrophages can release a variety of proinflammatory and fibrogenic mediators, such as IL-1, IL-3, IL-8, IL- 18, and TNF-α, able to attract circulating inflammatory cells and trigger and perpetuate the local inflammatory response. Inflammation is involved not only in the onset and progression of atherosclerotic process, but also in the complication of plaque rupture, probably due to the ability of activated macrophages and T cells to release hydrolytic enzymes, chemokines, cytokines, and growth factors. These factors lead to focal necrosis of the fibrous cap, which becomes highly susceptible to rupture, whereas the macrophage production of procoagulant mediators, such as tissue factor, triggers plaques thrombosis (29, 30).

Furthermore, endogenous products from damaged cells, such as cholesterol crystals, may trigger and amplify inflammatory response, through the activation of the Nlrp3 inflammasome, a complex of proteins involved in the proteolytic cleavage, maturation and secretion of IL-1 beta (31). IL-1 beta promotes smooth muscle cell proliferation, the recruitment of inflammatory cells and the increased production of other cytokines, such as IL-6, thus playing a crucial role in the inflammatory cascade (32).

Numerous inflammatory mediators have been extensively studied in the physiopathological processes of atherogenesis and plaque vulnerability. Elevated IL-6 levels have been associated with an increased risk of future myocardial infarction in apparently healthy men, thus supporting the role of inflammation in the early stages of atherogenesis. Furthermore, clinical studies have demonstrated the prognostic role of CRP and serum amyloid A levels in patients with coronary artery disease (CAD). The increase in these sensitive inflammation markers predicted an unfavorable outcome in patients with unstable angina (33–35).

Moreover, the large randomized controlled Canakinumab Anti-Inflammatory Thrombosis Outcomes Study (CANTOS) emphasized the role of IL-1b in cardiovascular risk and outcome, demonstrating that its inhibition with canakinumab significantly reduces the recurrence of new cardiovascular events in subjects with stable CAD and persistent increase in CRP (36). The use of Canakinumab has been also associated with a reduction in IL-6, CRP and fibrinogen circulating levels in patients with high vascular risk (37).

Inflammation plays a crucial role, not only in the pathophysiology of CAD, but also in the pathogenesis of aortic stenosis (AS). Interestingly, age, smoking, hypertension, hypercholesterolemia, obesity and diabetes, which are traditional risk factors for atherosclerosis, are also associated with development and progression of AS. Indeed, AS is a progressive and active process that shares many physio-pathological aspects with vascular atherosclerosis, such as endothelial dysfunction, lipid infiltration, oxidative stress, and activation and penetration of inflammatory cells into the endothelium of the aortic valve leaflets.

Secretion of the proinflammatory cytokines, IL-1β, TNF-a, IL-6, IL-8, insulin-like growth factor 1, TGF-β, promotes osteogenic differentiation of resident interstitial valve cells, leading to bone deposition and calcification (38).

More and more evidences are also accumulating on the role of inflammation in the pathogenesis of atrial fibrillation (AF). Histological analysis of the atria of AF patients revealed infiltration of lymphomononuclear cells and necrosis of adjacent myocytes (39).

Several inflammatory pathways may contribute to structural remodeling of the atria by modulating calcium homeostasis, cardiomyocyte apoptosis, and fibrosis. Furthermore, AF itself can induce inflammation that further improves atrial remodeling, activating a vicious cycle that perpetuates and increases the severity of the arrhythmia (40). Indeed, higher levels of inflammatory markers, such as IL-6, IL-8, and TNF, have been described in AF patients compared with subjects in sinus rhythm (41). The association between elevated CRP levels and the presence of AF has also been reported; furthermore, an increase in baseline CRP levels seems to predict the development of new onset AF. Overall, these data confirm the central role of persistent inflammatory state in the development of AF (42, 43).

Many lines of evidence demonstrate the involvement of inflammation also in development and progression of heart failure (HF). Elevated circulating levels of pro-inflammatory cytokines in HF patients compared with healthy individuals, have been reported, thereby revealing a potentially important role for the innate and adaptive immune system in the pathogenesis of the disease (44, 45).

Immune systems activation in HF comprises non-cellular and cellular components, including macrophages, mast cells, B cells, and T cells.

The hyperactivity of the sympathetic nervous system (SNS) and of the renin-angiotensin-aldosterone system (RAAS), that occur in HF, promote the activation of inflammatory cells, such as macrophages, T cells and B cells, as well as the loss of cardiomyocytes. In turn, myocardial damage acts as a trigger to support and amplify the inflammatory response in the heart (46).

The key role of inflammation in the pathogenesis of HF is also supported by histological studies of specimens of heart from HF patients, that revealed infiltrates of activated T lymphocytes, monocyte, macrophages and natural killer cells (47).

HF with preserved ejection fraction (HFpEF) also recognizes an inflammatory pathogenesis. HFpEF is a very challenging syndrome, very common in elderly patients, especially in the presence of multiple comorbidities, such as chronic kidney disease, chronic obstructive pulmonary disease, sleep disordered breathing, diabetes mellitus, sarcopenia, that can significantly contribute to a systemic proinflammatory state. All these chronic diseases are associated with high plasma levels of cytokines and inflammatory mediators, such as IL-6, TNF-a, soluble ST2 (sST2) and pentraxin, which promote endothelial dysfunction and the expression of VCAM and E-selectin, which in turn result in the migration and activation of monocytes in the subendothelium and the release of transforming growth factor (48). The conversion of fibroblasts into myofibroblasts determines the deposition of collagen in the interstitial space and the remodeling of the extracellular matrix. Furthermore, oxidative stress and the production of ROS result in reduced bioavailability of nitric oxide in cardiomyocytes with consequent lower activity of soluble guanylate cyclase (sGC) and consequently lower concentration of cyclic guanosine monophosphate and reduced activity of protein kinase G. The result is the induction of cardiomyocyte hypertrophy. Both hypertrophic cardiomyocytes and interstitial fibrosis, driven by inflammation, contribute to high diastolic stiffness of the left ventricle (LV) and to onset of HF (48).

## Epicardial Adipose Tissue in Elderly

Accumulating evidence strongly support the role of structural and functional changes of EAT in the pathogenesis of various cardiovascular diseases.

EAT is the visceral fat depot of the heart. It covers 80% of the heart's surface, thereby contributing for the 20% to the total heart weight. It is located between the visceral pericardium and the myocardium, lodges in the atrioventricular and interventricular furrows and along the major branches of the coronary arteries. It is a distinct tissue from the pericardial fat, while shares embryological origins and morphological aspects with visceral fat, both originating from mesodermal cells. EAT is mainly composed of small adipocytes, but also stromo-vascular and immune cells, and histological analysis also demonstrated the presence of ganglia and intercommunicating nerves, and variable degrees of leukocyte accumulation, with T lymphocytes, macrophages and mast cells (49).

Human EAT shows the molecular signature of a beige fat depot in middle-aged patients. These special features might portray stages in the trans-differentiation of the EAT from brown adipose tissue to white adipose tissue (50).

The anatomical closeness to the myocardium is one of the most interesting features of EAT; the two tissues are anatomically and functionally contiguous, without dividing boundaries, and share the same microcirculation. These aspects underlie the strong interaction between EAT and myocardium.

EAT is a metabolically very active tissue and performs numerous functions, through mechanical, vasocrine and paracrine actions. Epicardial fat, by surrounding coronary arteries, provides mechanical support and restricts their movement, thus protecting them from the tensions and torsion induced by the arterial pulse wave and cardiac contraction (51).

In physiologic conditions, EAT acts like brown adipose tissue, thereby having thermogenic function, and represents a local storage for FFAs. Thus, on the one hand, it ensures protection of cardiomyocytes and coronary arteries from toxic exposure to high circulating levels of FFAs; on the other hand, this deposit represents a direct source of FFAs, readily available for cardiomyocytes to generate energy under conditions of increased metabolic demand. Furthermore, EAT is a tissue with relevant endocrine properties, capable of producing and secreting antiatherogenic and anti-inflammatory cytokines. The anatomical contiguity to the myocardium and the sharing of the same microcirculation allow EAT to perform not only endocrine actions but also paracrine effects, directly disseminating cytokines and FAAs into the myocardium and coronary lumen. Among the EAT-released adipocytokines, there are adiponectin, adrenomedullin and omentin, which allow the control of vascular tension, by promoting vasodilatation, and hinder vascular remodeling (52).

Adrenomedullin also has antioxidant and antiapoptotic properties, inhibits the migration and proliferation of vascular smooth muscle cells and contributes to cardiac output, by increasing the availability of calcium for cardiomyocytes. Adiponectin, instead, is known to be an anti-inflammatory cytokine involved in the regulation of glucose and lipid metabolism: the increase in adiponectin levels is associated with an increase in insulin sensitivity, a reduction in the hepatic and muscular triglyceride content and in circulating FFAs levels (49).

In case of changes in the local microenvironment, the positive and beneficial effects of EAT can turn into harmful effects. This switch occurs in various pathological conditions, such as obesity, diabetes, and vascular diseases, which favor the shift of the EAT phenotype and secretome toward a pro-inflammatory, profibrotic and pro-atherosclerotic profile. In these conditions, an increase in the EAT thickness occurs, accompanied by a greater inflammatory and immune infiltrate, consisting of dendritic cells, T and B lymphocytes, macrophages and eosinophils and by an increase in production and secretion of cytokines and pro-inflammatory mediators, such as IL-1β, IL-6, TNFα, MCP-1, resistin and visfatin. Furthermore, in addition to an increase in the number of infiltrating macrophages, a cellular polarization is observed in the thicker and dysfunctional EAT. It consists in a phenotype shift of anti-inflammatory macrophages M2 toward the pro-inflammatory macrophages M1 and seems to correlate to the levels of EAT-derivated inflammatory cytokines (53).

Aging is one of the determining factors of the EAT amount. In subjects over 65 years of age, a mean total EAT amount of 22% greater compared to young subjects has been reported, thus suggesting an increase in epicardial fat with age (54). In patients without metabolic syndrome the presence of EAT ≥ 5 mm significantly increased with age (55). Similary, Silaghi et al. (56) described age as one of the main covariates associated with EAT extent, together with waist circumference and myocardial hypertrophy. Interestingly, Guglielmini et al. (57) reported that EAT depots appear to be more strongly associated with age than waist circumference or body mass index (BMI).

Human and animal studies demonstrated that aging is accompanied by increasing changes in body mass and fat redistribution and has a profound impact on EAT phenotype. Epicardial fat of old rats exhibited significantly lower levels of adiponectin and an increase in IL-6, compared to young rats (58). A population of 120 patients has been enrolled by Karadag et al. to examine the correlation between anthropometric values and EAT in young and old subjects. Simple regression analysis revealed statistically significant positive correlation between EAT and age, waist circumference and thigh circumference. Importantly, the effect of age and waist circumference on EAT continued to be statistically significant also on multivariate regression analysis. Interestingly, a statistically significant correlation between fasting insulin, insulin resistance and EAT was observed only in the geriatric group. This data, probably influenced by the higher incidence of insulin resistance with advancing age, highlights the importance of epicardial fat assessment in estimating the cardiometabolic risk of elderly patients (59).

The recognition of EAT as a marker of visceral adiposity and cardiometabolic risk is also confirmed by a study conducted on geriatric population, that identified obesity, HOMA index, fasting plasma glucose, weight, total and LDL cholesterol levels as determinants of EAT amount (60).

Other authors evaluated the relationship between visceral fat, hepatic steatosis, and metabolic syndrome occurrence in the elderly. Abdominal fat thickness and EAT thickness resulted to be greater in old patients with metabolic syndrome compared to non-metabolic syndrome patients. It has been observed an association between both increased abdominal fat thickness and EAT thickness with hepatic steatosis but only EAT resulted to be strongly correlated to metabolic syndrome (61).

In elderly patients with isolated severe calcific AS the increased EAT thickness, measured by echocardiography, correlates with levels of EAT secreted inflammatory and pro-atherogenic mediators, such as IL1β, IL-6, MCP-1, and TNF-α. These results support the thesis of an involvement of cardiac visceral fat in inflammatory and atherogenic phenomena that promote the development of cardiovascular diseases in the elderly and underlie the potential clinical utility of routinely EAT assessment (62).

Overall, the cytokines produced by EAT may contribute to the systemic inflammatory state, which in turn can promote the accumulation of EAT, inducing local and systemic inflammation and organ dysfunction. Thus, a continuous interlink is established between EAT, systemic inflammation and cardiovascular disease (9).

## Eat and Coronary Artery Disease

The central role of inflammation in endothelial dysfunction and cardiovascular diseases has been widely described (63, 64). Obesity promotes systemic inflammation and is recognized as a major cardiovascular risk factor (65, 66). Of note, the cardiovascular risk in obese subjects is mainly associated with visceral rather than subcutaneous adiposity (67). A dysfunctional perivascular adipose tissue, also through the down- and up-regulation of adiponectin and leptin, favors many features of atherosclerosis (68). A greater deposit of VAT has been observed in non-obese patients with CAD (69). Interestingly, in CAD patients, VAT has been associated with the presence of multivessel disease (70) and the risk of coronary plaque progression (71).

Importantly, obesity and the consequent chronic systemic inflammation result to be associated to a significant increase of the EAT amount (72). Therefore, several researchers have investigated the role of EAT in the development and progression of CAD. Ahn et al. (73) conducted a study on 527 patients undergoing their first coronary angiography, to explore the relationship between EAT, CAD risk factors and the extent of coronary atherosclerosis. EAT thickness was correlated with age and abdominal VAT and, above all, a greater EAT thickness was observed in CAD subjects compared to controls. Accumulation of EAT also correlated with the extent and activity of CAD, being thicker in patients with unstable angina than those with stable angina or atypical chest pain.

These data agree with the results of another study, that showed higher EAT thickness in patients with angiographic CAD. The authors also reported the correlation between EAT increase and number of vessels with >50% diameter stenosis (74).

The positive association between EAT volume and CAD burden is supported by studies conducted on pig models of CAD. In these animal studies, surgical resection of EAT depot decreased the progression of CAD, thus suggesting that EAT may contribute locally to CAD and exacerbate coronary atherosclerosis (75, 76).

In vulnerable plaques 18-fluorodeoxyglucose (^18^F-FDG) imaging detect higher macrophage infiltrates, evidenced by an increased standardized uptake value (SUV) (77). Positron emission tomography (PET)-CT was performed in a prospective study conducted on patients with low or intermediate risk of acute coronary syndrome to investigate whether the inflammatory activity of pericoronary adipose tissue could be associated to plaque composition. Interestingly, the maximum SUV of ^18^F-FDG was measured in the fat surrounding the coronary arteries and correlated with plaque burden and necrotic core component of coronary plaque (78).

The prominent impact of EAT inflammation on CAD risk, has been confirmed by studies that compared expression of inflammatory mediators in epicardial and subcutaneous adipose stores in patients undergoing coronary artery bypass graft surgery (CABG). A greater inflammatory cell infiltrates has been shown in EAT with respect to the subcutaneous adipose tissue, accompanied by a marked increase of EAT-derived inflammatory mediators, such as IL-1β, IL-6, MCP-1, and TNF-α, and their messenger RNA (79).

The macrophage polarization observed in EAT, characterized by a relative increase of inflammatory M1 macrophages and a relative decrease of anti-inflammatory M2 macrophages, is considered one of the underlying mechanisms of increased pro-inflammatory mediators in EAT of CAD patients. Importantly, it has been demonstrated a positive correlation between the M1/M2 macrophages ratio and the severity of CAD, thereby supporting the crucial pathological role of this phenomena in the coronary atherosclerotic process (53, 80).

Increased macrophage infiltrate and macrophage polarization are associated with increased production and secretion of inflammatory mediators (81) and may also explain the increased expression and secretion of resistin in EAT of patients with acute coronary syndrome, compared to patients with stable CAD or subjects without CAD (82). It has also been recently shown that plasma resistin levels are predictive of mortality in patients with acute myocardial infarction (83). Resistin seems to promote inflammation and atherogenesis by counteracting vasodilation and increasing the expression of adhesion molecules on endothelial cells (84, 85).

Similarly, animal studies on pig models of metabolic syndrome have indicated perivascular adipose tissue-derived leptin as a potential contributor to coronary atherogenesis. The increased leptin in EAT surrounding coronary arteries exacerbates endothelial dysfunction by reduction of nitric oxide production via protein kinase C-beta phosphorylation of endothelial nitric oxide synthase (86).

The prospective EPICHEART study evaluated proteomic EAT profile in 574 patients with severe AS and referred to cardiac surgery. EAT volume quantified by CT was associated with higher CT-derived Agatston coronary calcium score. Furthermore, EAT exhibited a pro-calcifying proteomic profile in CAD patients, consisting in upregulation of annexin-A2 and downregulation of fetuin-A. Annexin-A2 protein levels in EAT samples were also positively correlated with agatston coronary calcium score, suggesting that EAT might orchestrate pro-calcifying conditions in the late phases of CAD (87).

These results are consistent with other clinical studies documenting an increase in the volume of perivascular EAT as a risk factor for coronary atherosclerosis (88) and attributing a crucial role to the local EAT release of potentially atherogenic adipokines in the development and progression of CAD (89, 90).

The pro-atherogenic properties of EAT in CAD are determined not only by an increase in pro-inflammatory cytokines but also by a reduction in anti-inflammatory mediators. In detail, lower levels of EAT-derived adiponectin were found in CAD patients. Adiponectin is known for its vasodilatory, antiaterogenic, anti-inflammatory, antioxidant properties which it carries out through the inhibition of the expression of IL-8 by endothelial cells, the stimulation of the anti-inflammatory cytokine IL-10 and the inhibitor tissue of MMP-1 in macrophages. Thus, the imbalance between pro and anti-inflammatory cytokines confers EAT a pro-inflammatory and pro-atherosclerotic phenotype (90). Accordingly, acute coronary syndromes are characterized by an imbalance between EAT secreted IL-1ß and its receptor antagonist (IL-1ra) (91).

## Eat and Aortic Stenosis

Calcific AS is a progressive and active process with multifactorial pathogenesis that includes biological mechanisms promoting the differentiation of resident valvular interstitial cells in osteoblast-like cells, thus leading to leaflets calcification. These events are mediated by proinflammatory cytokines such as IL-1β, IL-6, IL-8, insulin- like growth factor 1, TNF-a, TGF-β, mainly secreted by macrophages and activated T lymphocytes penetrating the endothelium of aortic valve leaftlets (92, 93).

In the last decades, several evidence recognized obesity and metabolic syndrome as relevant risk factors for AS, as well as for atherosclerosis, and highlighted the pathogenetic role of VAT in the development and progression of AS, thus identifying VAT as an independent risk factor for this valve disease (94).

Obesity and metabolic syndrome have been associated with the risk for both development and progression of aortic valve calcification, in the MESA (Multi-Ethnic Study of Atherosclerosis) and ASTRONOMER (Aortic Stenosis Progression Observation Measuring Effects of Rosuvastatin) studies, respectively (95, 96).

VAT production of inflammatory cytokines and ROS support its role in the pathogenesis of AS (97).

The hypothesis of a pro-inflammatory activation of EAT in AS patients is supported by several evidence which show that EAT amount is increased in these patients and its secretome is particularly rich in inflammatory mediators. It has been shown that EAT thickness correlates significantly with the levels of numerous secreted proinflammatory and pro-atherogenic cytokines, such as IL-6, TNF-α, MCP-1, IL-1β. Interestingly, this correlation was not found with plasma levels of the same mediators, which were similar in patients with and without AS, further reinforcing the hypothesis of the role of local inflammation (62).

The observation of increased EAT thickness in patients with severe AS was also confirmed by a retrospective study including a cohort of 200 consecutive patients with severe AS and 200 matched patients without AS. The logistic regression analysis showed that an increase in EAT by one standard deviation was associated with a two-fold increased occurrence of AS. Of note, EAT thickness was significantly associated with severe AS, independently from age, gender and cardiovascular risk factors (98).

The potential action of EAT in promoting inflammatory and atherosclerotic changes in the aortic valve appears to occur in the early stages of the disease. In this regard, a clinical study evaluated the correlation between EAT thickness and aortic valve sclerosis, by involving 225 patients who were admitted for coronary angiography due to new-onset angina. Transthoracic echocardiography was performed to assess both EAT thickness and the average aortic valve sclerosis score index. The authors reported that patients with an EAT thickness ≥7 mm were older, with more frequent hypertension and hyperlipidemia, and showed greater LV hypertrophy and a higher average aortic valve sclerosis score index, thus suggesting an association between EAT and aortic leaflets calcification already in the early stages of the disease (99).

Starting from the assumption of anti-inflammatory and anti-atherosclerotic properties of statins, several studies have suggested EAT as a potential new therapeutic target for statin therapy. In a population of elderly patients with calcific AS, statin therapy was significantly associated to a reduction of echocardiographic EAT thickness, that was paralleled by an attenuation of EAT inflammatory profile. *In vitro*, atorvastatin showed a direct anti-inflammatory effect on EAT, thereby indicating that statin may directly modulated EAT secretory profile (100).

However, there are conflicting data on the potential beneficial effect of statins in slowing the progression of AS. Rajamannan et al. developed an experimental hypercholesterolemic rabbit model and showed a proliferative atherosclerosis-like process associated with the transformation to an osteoblast-like phenotype in the aortic valve. Of note, they demonstrated that atorvastatin could inhibit these processes, by counteracting lipids deposition and oxidative stress observed in the early stages of degenerative calcified aortic disease (101).

In humans, the first prospective study showing a positive effect of statin therapy for AS progression, has been conducted by Moura et al., by evaluating 121 consecutive AS patients treated with and without rosuvastatin. Statin therapy was associated with a slowing of the hemodynamic progression of the disease (102).

However, three randomized controlled trials disagreed with these results. In fact, in patients with mild to moderate AS, the use of atorvastatin, rosuvastatin and simvastatin plus ezetimibe significantly reduced serum LDL cholesterol levels but was not associated with a beneficial effect on the progression of AS. Too late initiation of treatment could be the possible explanation for the statins failure to influence AS progression (103–105).

Long-term controlled studies enrolling patients with less advanced AS are needed to establish the true effect of statins in influencing AS progression and to better understand the possible influence of statin therapy on EAT activity.

## Eat and Atrial Fibrillation

Numerous studies (106, 107) have reported the association between obesity and AF, and more recently, growing evidence explored the link between EAT and onset, severity and recurrence of AF. This relationship is the result of complex crosstalk between EAT and the neighboring atrial myocardium (108).

In a CT analysis of 3,217 individuals from the Framingham Heart Study, EAT volume was associated with prevalent AF, indipendently by traditional AF risk factors, including BMI (109).

EAT amount correlates not only with the presence of AF but also with AF severity and progression. Tsao and colleagues used CT images to quantify epicardial fat surrounding the atrium and demonstrated significantly increased volume of EAT surrounding the left atrium in AF patients and a tendency for persistent AF patients to have larger volumes than those with paroxysmal AF. There was no association between BMI and AF severity (110).

A recent meta-analysis reinforced the association between EAT amount and atrial arrhythmias, showing that EAT volume is higher in patients with persistent and paroxysmal AF than in healthy subjects. Interestingly, a significant increase in EAT was also found in patients with persistent AF compared to patients with paroxysmal AF, thus supporting the association between EAT amount and AF severity (111).

Several pathogenetic mechanisms have been proposed to explain the implication of EAT in AF onset and progression. EAT has been proposed as a potentially important factor involved in structural and electric atrial remodeling. Given the proximity of EAT to the underlying myocardium, it can directly influence atrial reeling by penetrating the myocardium and generating atrial fatty infiltrates, that may alter the atrial electrophysiological properties by determining the loss of side-to-side cells connection. The result is the creation of circuits that compromise the propagation of the depolarizing wave and generate the return phenomena (112, 113).

EAT can also indirectly promote atrial remodeling and alter atrial electrophysiological properties, by acting as a source of paracrine modulators of myocardial inflammation and oxidative stress.

EAT releases pro-fibrotic factors, such as TGF-β1, the adipokine activin A, a member of the TGF-β superfamily and MMP2, MMP7, key regulators of extra-cellular matrix activity, that are up-regulated during AF, thereby contributing to atrial collagen deposition, fibrosis and remodeling (114–116).

EAT is also a relevant source of inflammatory cytokines, such as IL-1β, IL-6, IL-8, and TNFα, that may have local pro-inflammatory effects on the adjacent atrial myocardium that facilitate arrhythmogenesis.

Several studies have associated the increase in inflammation markers, such as CRP, IL-6, IL-8, IL-1β, and TNF-α, with the presence and severity of AF (42, 117), and EAT seems to be a major source of inflammation in patients with AF. Mazurek et al. examined EAT inflammatory activity using FDG-PET/TC in patients with AF compared to non-AF controls subjects and showed that EAT inflammatory activity was increased in AF patients. Interestingly, EAT tracer uptake was greater than subcutaneous or other visceral adipose tissue depots, suggesting a marked inflammatory activity of EAT that could contribute to the development of AF (118).

EAT can contribute to the pathogenesis of AF also through the induction of oxidative stress. EAT is a source of ROS, that exert detrimental local effects on atrial myocardium. The role of oxidative stress from EAT in the genesis of AF is confirmed by experimental data showing that ROS inhibition by antioxidants attenuates atrial remodeling in animal models (119).

EAT could promote AF also through the production of an enzymatic protein, known as aromatase, that acts converting androgens into estrogens, and which seems to play an important role in modulating electromechanical properties, with consequent susceptibility to atrial arrhythmias. Aromatase is abundantly expressed in adipose tissue as well as in the myocardium and in EAT and its expression is remarkably upregulated with aging. In experimental models, EAT aromatase levels were higher in aged than in young animals and its estrogen conversion capacity resulted to be significantly enhanced with the increase in EAT amount, suggesting an association between EAT, aromatase estrogenic capacity and atrial arrhythmogenicity (120).

In addition to these pathogenetic mechanisms, it has been hypothesized that EAT could function as a trigger at the pulmonary veins level and/or other sites. An increased EAT volume may alter the function of ganglionated plexi, located near the pulmonary veins, leading to spontaneous, rapid and repetitive electrical activity that can promote AF (121).

Ganglionated plexi have been identified also in EAT, and their activation can cause autonomic nervous system stimulation resulting in shortening of action potential duration and increasing in calcium transient in the atrial myocardium, thereby contributing to arrhythmogenesis. These hypotheses are supported by the efficacy of botulinum toxin injection into EAT in suppressing atrial tachyarrhythmia, potentially through the inhibition of ganglionated plexi (122).

EAT, through the local production and secretion of catecholamines, may directly contribute to an increased sympathetic tone and to a sympatho-vagal imbalance, thus promoting atrial arrhythmias. Interestingly, EAT has a higher adrenergic activity compared to subcutaneous adipose tissue, demonstrated by the increased catecholamine levels and expression of catecholamine biosynthetic enzymes, thus supporting a potential role of EAT in arrhythmogenesis (123).

## Eat and Heart Failure

The role of EAT in the pathogenesis and progression of HF has been described by several studies and includes complex pathophysiological mechanisms. EAT can sustain the processes underlying HF by regulating myocardial remodeling, insulin resistance, and RAAS. Adipokines and inflammatory cytokines secreted by EAT could mediate these actions. In fact, proinflammatory polarization of EAT is common in patients with HF and consequent changes in the EAT secretome promote myocardial dysfunction, by exacerbating inflammation and cardiac fibrosis, thus favoring arrhythmogenesis and HF progression (124).

EAT obtained from obese HFpEF patients showed a marked increase in inflammatory infiltrate, consisting in T lymphocytes and M1 macrophages, supporting the potential role of local inflammation in the pathogenesis of disease (125).

It has been shown that in obese individuals the increase in EAT volume was significantly associated with impaired myocardial microcirculation, abnormal cardiac diastolic properties, increased vascular stiffness and left atrium dilatation, all typical features of HFpEF (126, 127).

The leptin-aldosterone-neprilysin axis appears to be the main pathway linking EAT dysfunction to the development and progression of HF (128). EAT contributes to increased circulating levels of leptin, promoting systemic inflammation, thereby negatively affecting the heart and other visceral organs (128–130).

Leptin can stimulate adrenergic- and angiotensin-dependent mechanisms and represents the major stimulus to the production of aldosterone, thereby being responsible for the excessive mineralocorticoid receptor signaling observed in HF (131, 132). Leptin is linked to the accumulation and dysfunctional biology of EAT which may directly lead to inflammation, microcirculatory abnormalities, and fibrosis of the underlying myocardium (133, 134). Moreover, the activation of leptin-aldosterone-neprilysin axis, which results in sustained increased levels of aldosterone and neprilysin, further promotes EAT accumulation and inflammation (135, 136).

The local activation of the RAAS promotes lipotoxicity, reduced mitochondrial respiration and insulin resistance, thus contributing to HF. The detrimental effects of RAAS activation on heart function have been observed both in humans and in experimental models (125).

Protracted exposure to inappropriately elevated aldosterone levels causes significant changes in LV structure and function. Of note, hyperaldosteronism promotes sodium retention, increases cardiac filling pressures, induces worse remodeling, and accelerates HF progression (137).

Angiotensin II stimulates the secretion of complement-C1q and TNF-related protein 1 (CTRP1), that is an adipokine associated with metabolic syndrome, adiponectin deficiency, platelet aggregation, athero-inflammation and hypertension. CTRP1 acts as a stimulating factor for endogenous aldosterone (138). In animal studies, proinflammatory cytokines including TNF-α and IL-1β caused elevations of CTRP1 levels in adipose tissue, indicating that CTRP1 expression may be associated with a low-grade chronic inflammation status in fat depots (139). Interestingly, Yang et al., showed that CTRP1 levels were increased both in the plasma and EAT of HF patients and proposed the CTRP1 involvement in the pathogenesis of HF by modulating IL-6 levels and aldosterone release (140).

EAT-derived miRNAs can also be involved in the pathophysiology of HF. In HF patients, increased secretion of several miRNAs was observed by EAT-resident macrophages, such as miR-21, which was upregulated in response to cardiac overload, and correlated with the TGF-β pathway causing hypertrophy and myocardial fibrosis (141).

Moreover, EAT-secreted MiR-17-5p is upregulated in obese subjects, increases adipogenesis, and has been described as a biomarker for HF (142). Similarly, miR-27 suppresses adipocyte differentiation and contributes to HF (143).

Cardiac SNS hyperactivity is associated to HF and results in sympathetic denervation of the heart. Several evidence indicate a possible contribute of EAT to SNS activation in HF (144). EAT may promote HF-related cardiac adrenergic derangement by stimulating central SNS activity through dysregulated adipokines production and secretion (145).

In patients with systolic HF, it has been demonstrated that EAT is a local source of catecholamine, and its increased thickness is associated to myocardial impaired autonomic function. Therefore, EAT seems to play an additive role in determining cardiac sympathetic denervation, in the context of HF-SNS hyperactivity (123). SNS activation in HF patients has been associated also with the prevalence of central sleep disordered breathing. Interestingly, EAT thickness resulted to be increased in these patients and correlated with increased circulating levels of norepinephrine and with the presence and the severity of sleep apneas (146, 147).

Overall, the evidence on the possible contribution of EAT to SNS activation in HF reinforces the potential negative role of EAT in the pathogenesis and progression of HF.

## Eat and Cardiovascular Outcome

Given the role of EAT in the pathophysiology of cardiovascular diseases, numerous studies have been conducted to explore the potential impact of EAT on cardiovascular outcome (Table 1).

**Table 1:** Associations between EAT and cardiovascular diseases.

**References**	**Patients source**	**EAT measure**	**Outcome**	**Association/Distribution**
Ahn et al. (73)	527 patients undergoing PCA for suspected CAD	EAT thickness ≥ 3 mm in diastole at echocardiography	CAD (stenosis ≥50%)	OR: 3.36 (95%CI: 2.2 - 5.2)
Picard et al. (74)	970 patients undergoing PCA for suspected CAD	EAT thickness EAT ≥2.8 mm at CT	CAD (stenosis ≥50%)	OR: 1.67 (95%CI: 1.23 - 2.26)
Tanindi et al. (148)	200 CAD patients (stable angina pectoris or acute coronary syndrome)	EAT thickness ≥7 mm in end-systole at echocardiography	- Cardiovascular death - AMI	HR: 1.9 (95%CI: 0.4-8.3) HR: 2.4 (95%CI: 0.6-10.0)
Mirdamadi et al. (149)	78 CAD patients referred to CABG	Intraoperative EAT thickness measure ≥6.5 mm	In-intensive care unit complications after CABG	OR: 1.33 (95%CI: 1.04–1.71)
Nelson et al. (150)	356 subjects referred to cardiovascular risk assessment	EAT thickness ≥ 5 mm in end-diastole at echocardiography	Coronary calcium score	HR: 2.26 (95%CI: 1.44–3.53)
Jeong et al. (89)	203 CAD patients undergoing PCA	EAT thickness ≥7.6 mm in end-diastole at echocardiography	CAD (stenosis ≥ 50%)	OR: 10.53 (95%CI: 2.2–51.2)
Parisi et al. (62)	95 severe AS patients referred to AVR vs. 44 healthy subjects	EAT thickness in end-systole at echocardiography	Association to AS	9.85 ± 2.78 mm (AS) vs. 4.91 ± 1.27 mm (controls); *p* < 0.0001)
Mahabadi et al. (98)	200 severe AS patients vs. 200 matched non-AS patients	EAT thickness increase of 1 SD at echocardiography	Occurrence of AS	OR: 2.10 (95%CI: 1.65-2.68)
Eberhard et al. (151)	503 AS patients referred to TAVR	EAT volume > 125 mm^3^ at multi-detector CT	All cause 3-year mortality after TAVR	HR: 2.27 (95%CI: 1.44–3.57)
Davin et al. (152)	118 patients with moderate or severe AS	Indexed EAT volume >60 ml/m^2^ at CMR	Adverse cardiovascular outcome	Indexed EAT volume > 60 ml/m^2^ vs. ≤ 60 ml/m^2^; *p*= 0.0088
Thanassoulis et al. (109)	3217 individuals from the Framingham Heart Study	EAT Volume increase of 1 SD at multidetector CT	Prevalence of AF	OR: 1.28 (95%CI: 1.03-1.58)
Tsao et al. (110)	68 AF patients vs. 34 non-AF controls	EAT Volume at multidetector CT	- AF occurrence	EAT Vol: 29.9 ± 12.1 (AF) vs. 20.2 ± 6.5 cm^3^ (non-AF); *p* < 0.001
			- AF recurrence after ablation	EAT Vol: 26.8 ± 11.1 (AF) vs. 35.2 ± 12.5 (non-AF): *p*= 0.007
Chu et al. (153)	190 persistent AF patients	EAT thickness ≥ 6 mm in end-diastole at echocardiography	Adverse cardiovascular events	OR: 1.224 (95%CI: 1.096-1.368)
Maeda et al. (154)	218 AF patients undergoing AF ablation	EAT Volune Index cut off ≥116 mL/m^2^ at multidetector CT	post-ablation recurrence of AF	HR: 1.02 (95%CI: 1.00-1.03)
Parisi et al. (155)	69 sistolic HF patients referred to ICD	Echocardiographic EAT thickness increase of 1 SD	Composite clinical and arrhythmic outcome	HR: 1.16 (95%C.I: 1.08–1.24)
Wu et al. (124)	58 systolic HF patients, 63 HFpEF patients, 59 non-HF patients	EAT volume at CMR	- HF patients vs. HFpEF patients vs. non-HF patients	Indexed EAT vol: 27.0 (22.7-31.6) vs. 25.6 (21.4-31.2) or 24.2 (21.0-27.6) mL/m^2^; *p* < 0.05
			- Cardiac fibrosis	r: 0.49; 95% CI: 0.12-0.86, *p* < 0.01
Nakanishi et al. (126)	372 patients undergoing CFR examination	Increase EAT volume of 10 ml at multi-detector CT	Deterioration of LV diastolic function	OR: 1.11 (95%CI: 1.02-1.21)

*EAT, epicardial adipose tissue; PCA, percutaneous coronary angiography; CAD, coronary artery disease; AS, aortic stenosis; AF, atrial fibrillation; HF, heart failure; AVR, aortic valve replacement; TAVR, transcatheter aortic valve replacement; ICD, implantable cardioverter defibrillator; CABG, coronary artery bypass graft surgery; CFR, coronary flow reserve; LV, left ventricle; AMI, acute myocardial infarction; CT, computed tomography; CMR, cardiac magnetic resonance; SD, standard deviation; 95%CI, 95% confidence interval; OR, odds ratio; HR, hazard ratio; r, correlation (Pearson or Spearman)*.

Tanindi et al. showed that CAD patients with EAT thickness more than 7 mm are at higher risk of myocardial infarction and cardiovascular death (148).

EAT thickness proved to be also useful for predicting in-intensive care unit complications after CABG surgery, such as AF onset, prolonged inotrope use, and fever (149).

Eberhard et al. evaluated the prognostic value of CT-EAT volume in 503 patients with severe AS undergoing transcatheter aortic valve implantation (TAVI). In this population, EAT volume was independently associated with all-cause mortality at 1, 2, and 3 years after TAVI. Therefore, these authors proposed the pre-TAVI assessment of EAT volume as a relevant prognostic factor for risk stratification of AS patients (151).

Similarly, Davin et al. investigated the contribution of EAT and late gadolinium-enhancement, quantified by CMR, on AS patients outcome. They showed that EAT volume predicts adverse outcome in AS asymptomatic patients, thus EAT volume assessment may improve the risk-stratification of asymptomatic AS patients (152).

The association between EAT and worse prognosis in AS patients probably lie in the potential EAT-related unfavorable LV-remodeling in response to the chronic pressure overload of LV.

Initially, LV hypertrophy is an adaptive phenomenon allowing the heart to maintain adequate cardiac output, by overcoming the obstacle of valve stenosis. However, over time it becomes a maladaptive phenomenon and evolves toward diastolic dysfunction, leading to HF and poor prognosis (156).

Numerous evidence support the role of EAT in promoting myocardial hypertrophy. Coisne et al. showed an independent and significative association between EAT echocardiographic thickness and pathological LV remodeling in AS patients. The intense metabolic and pro-inflammatory activity of EAT could account for this association (157). These data confirm the previous hypothesis of Iacobellis et al. (158) who found for the first time that an increase in epicardial fat, assessed by echocardiography, is significantly related to an increase in LV mass.

EAT echocardiographic thickness has been also identified as a marker of cardiac adrenergic derangement that is strongly correlated with adverse prognosis in HF patients. EAT thickness proved to be an independent predictor of cardiac sympathetic innervation evaluated by ^123^I-metaiodobenzylguanidinescintigraphy (^123^I-MIBG) (123), and, in high-risk patients, is associated to both clinical and arrhythmic outcome (155). Furthermore, in HF patients, echocardiographic EAT thickness was found higher in presence of sleep-disordered breathing and progressively and significantly increased with the severity of the sleep apneas (147), that are associated with a greater SNS activation (159) and a worse outcome in patients with HF (160). This association supports the hypothesis that EAT abnormalities might represent a novel pathophysiological link between sleep-disordered breathing, SNS hyperactivation and prognosis in HF patients.

The correlation between EAT and cardiovascular outcome has been described also in AF patients. Tsao et al. described an independent association between increased EAT around the left atrium and stroke in AF patients. EAT amount was correlated with the contractile dysfunction of the left atrium and the circulatory stasis of the atrial appendage, two important risk factors for AF-related stroke (161). Similarly, Chu et. Al reported the negative prognostic role of EAT thickness in AF patients. EAT was associated with adverse cardiovascular outcome, being correlated to cardiovascular mortality, hospitalization for HF, myocardial infarction, and stroke. Moreover, adding EAT thickness to a predictive model containing CHA2DS2-VASc score, left atrial volume, and LV systolic and diastolic function, EAT significantly improved the risk stratification (153). Interestingly, EAT thickness is positively correlated with CHA2DS2-VASc risk score, thus confirming the association between EAT, stroke and adverse cardiovascular outcomes in AF patients (162). Maeda et al. showed a close correlation between EAT and recurrence of post ablation AF, that was more frequent in patients with higher EAT amount, measured using CT. These authors demonstrated that an EAT volume index cutoff ≥116 mL/m^2^ may be useful for prediction of recurrent AF after catheter ablation (154). Another study showed that the EAT pro-arrhythmic influence is greater in the early post-ablation phase. EAT amount independently predicted early recurrence after AF ablation whereas it did not have an impact on late recurrence (163). According to these findings, a systematic review and meta-analysis that compared EAT amount between patients with and without AF recurrence showed that total and left atrial-EAT volumes, as well as EAT thickness, were higher in patients with AF recurrence (164).

Overall, these evidence indicate that EAT quantification can be used along with traditional predictors, as a new imaging marker to predict cardiovascular outcome.

## Eat Quantification

Several imaging methods have been used to provide a reliable quantification of EAT, such as echocardiography, CT and CMR (Table 1).

CMR imaging is considered the gold standard for the detection and quantification of EAT, providing an accurate and volumetric EAT measurement (165), especially once new CMR imaging method using the three-dimensional summation of slices approach has been developed (166).

The measure of absolute amount of EAT is obtained volumetrically in consecutive short-axis views by means of the modified Simpson's rule. In addition, maximum EAT thickness at the right ventricular free wall is measured in a transversal 4-chamber view and averaged in consecutive short-axis views covering the whole ventricle; the mean EAT thickness is calculated by averaging results from the long-axis and short-axis measurements. The results of these measurements appear to be consistent with the autopsy measurement of EAT thickness obtained in 200 human hearts at the ventrolateral edge of the right ventricle (167).

Despite the great diagnostic potential, CMR imaging is an expensive and time-consuming procedure, so it is hardly available routinely in clinical practice.

CT allows the measurement of the EAT with the higher spatial resolution as compared to echocardiography and CMR imaging, thus providing the best sensitive and accurate assessment of EAT thickness, volume and total area (168). The three-dimensional image reconstruction with multidetector-row CT further improves spatial resolution and the specificity and sensitivity of measurements (169). In the populations of the Framingham Heart Study and the Multi-Ethnic Study of Atherosclerosis, variable values of mean EAT volume detected by CT, ranging from 68 ± 34 mL to 124 ± 50 mL, have been reported (170, 171). In a cohort of 226 subjects with a low Framingham Risk Score referred to non-contrast cardiac CT for coronary calcium scoring, the value of 68.1 mL/m^2^ at CT has been identified as the 95th percentile of EAT volume indexed to body surface area. EAT volume exceeding this value seems to predict major adverse cardiovascular events (172). The reproducibility of volumetric quantification of EAT by cardiac CT appears to be superior to that of thickness and area measurements. However, volumetric assessment is time consuming, requires an advanced cardiac imaging workstation and qualified and experienced observers (168). Furthermore, the high cost and the exposure to ionizing radiation limit the use of this procedure in routine clinical practice.

Echocardiography allows the assessment of the EAT thickness in a safe, inexpensive and easily repeatable way, without the risk of exposure of the patient to ionizing radiations. Despite these intuitive advantages, it has some important limitations: it is an operator-dependent procedure and allows an accurate estimation of EAT thickness but not of EAT volume. In addition, a poor acoustic window, such as in obese or very thin subjects, can prevent optimal visualization of the EAT thickness or make it difficult.

Over the years, various authors have proposed different echocardiographic approaches for measuring epicardial fat, which differ both in the measurement site and in the phase of the cardiac cycle chosen for the EAT thickness assessment. Some authors have proposed the EAT measurement at end-diastole (73), to conform with other imaging techniques, such as CT and CMR; conversely, other authors recommend measuring EAT at end-systole (173), thus avoiding any EAT compression during diastole.

Iacobellis et al. described EAT as an echo-free or a hyperechoic space, if it is massive, visible from both parasternal longitudinal and transverse views, and suggested to measure the maximum EAT thickness in systole, between the right ventricular free wall and the parietal pericardium, as the average of three consecutive beats. In healthy people with body max index > 22 kg/m^2^, they showed a variability of EAT thickness values, between 1.8 and 16.5 mm (174). By using the same echocardiographic method, Malvazos et al. observed an EAT thickness of 6.5 ± 0.8 mm in obese patients, and of 1.3 ± 0.2 mm in healthy subjects, from both parasternal long- and short-axis views (175).

On the 2-dimensional echocardiography, EAT was described by Jeong et al. as an echo-free space in the pericardial layers (89) and its thickness was measured in more than 200 subjects referred to coronary angiography, at end-diastole, from the parasternal long-axis views, perpendicularly on the free wall of the right ventricle, for 3 cardiac cycles. The authors reported an average EAT thickness value of 6.4 mm (1.1–16.6 mm). Nelson et al. (150) also measured EAT thickness in asymptomatic subjects presenting for cardiovascular preventive care. The measurement was obtained on the free wall of the right ventricle in parasternal long-axis view, at end-diastole, perpendicular to the aortic annulus. They found a mean value of 4.7 ± 1.5 and suggested a cut-off EAT thickness value of 5 mm as marker of cardiovascular risk.

However, as known, EAT is localized between the myocardium and the visceral layer of the pericardium; therefore, some of these approaches are not faithful to its true anatomical site and can cause confusion between epicardial fat and pericardial fluid. Our group validated the measurement of EAT at the level of the Rindfleisch fold, between the free wall of the right ventricle and the anterior surface of the ascending aorta (173). In this pericardial recess, the space between the two pericardial layers is greater, so that EAT, not compressed by parietal pericardium, can expand and EAT can be visualized in its maximum thickness and directly measured. This approach is faithful to the true anatomical site of the EAT, located between the myocardium and the visceral layer of the pericardium, allows for direct visualization of the fat depot and its simple measurement. Furthermore, unlike other echocardiographic methods (176), this measurement seems to reflect the total amount of EAT, and well correlates with EAT thickness and volume at CMR, the current gold standard for EAT quantification.

Further studies are required on comparing and improving fat measurement techniques and methods, to reach a consensus on how to measure EAT, define normal reference values and introduce this measurement into clinical practice.

## Conclusions

Human aging is characterized by a state of chronic low-grade inflammation that significantly contributes to cardiovascular diseases and adverse outcomes in the elderly. The accumulation of visceral fat depots is accompanied by an increased inflammatory status and promotes the low-grade inflammation. A greater EAT amount, the visceral fat depot of the heart, is observed in the elderly. The anatomical features and biological function of EAT raised a growing interest on the potential role of EAT in cardiovascular diseases. Increased EAT is paralleled by an increased inflammatory status and a greater EAT secretion of inflammatory mediators and neuro-hormones, which may penetrate the myocardium and coronary vessels in a paracrine and vasocrine manner and express their toxicity in the neighboring tissues, thus contributing to development and progression of cardiovascular diseases. Accumulating evidence strongly support the correlation between EAT accumulation and a worse cardiovascular outcome.

Different imaging methods, such as echocardiography, CT and CMR, are available for EAT quantification in clinical practice, and EAT measurement has been proposed as useful marker for assessment of cardiovascular and metabolic risk. Further studies are needed to validate standardized measurement methods and to better define normal reference values of EAT, thereby leading to a routinely use of EAT volume/thickness in cardiovascular risk stratification.

## Author Contributions

MC and VP conceived the manuscript structure and wrote the manuscript with support from LP, PP, and VV. SC contributed to the writing and revision of the Aging and inflammation and Epicardial adipose tissue in elderly sections. PC and GC contributed to the writing and revision of the coronary artery disease and aortic stenosis sections. EA and VR contributed to the writing and revision of the atrial fibrillation and heart failure sections. EP and PF contributed to the writing and revision of the cardiovascular outcome section. VP and DL supervised other authors and contributed to the final version of the manuscript. All authors contributed to the article and approved the submitted version.

## Conflict of Interest

The authors declare that the research was conducted in the absence of any commercial or financial relationships that could be construed as a potential conflict of interest.

## Publisher's Note

All claims expressed in this article are solely those of the authors and do not necessarily represent those of their affiliated organizations, or those of the publisher, the editors and the reviewers. Any product that may be evaluated in this article, or claim that may be made by its manufacturer, is not guaranteed or endorsed by the publisher.
